# 

**Published:** 1891-10-31

**Authors:** 


					AND INVALIDS
I INFANTS
*70* TlDRWICH HOSfLTAJ.
.Ksaotr Wester* Infirmarx
WITH EXTRA NURSING SUPPLEMENT.
Saturday, October 31st, 1891

				

